# National routine adult immunisation programmes among World Health Organization Member States: an assessment of health systems to deploy COVID-19 vaccines

**DOI:** 10.2807/1560-7917.ES.2021.26.17.2001195

**Published:** 2021-04-29

**Authors:** Sarah R Williams, Amanda J Driscoll, Hanna M LeBuhn, Wilbur H Chen, Kathleen M Neuzil, Justin R Ortiz

**Affiliations:** 1 Division of Pulmonary and Critical Care Medicine, University of Maryland School of Medicine, Baltimore, Maryland, United States; 2 These authors contributed equally to this manuscript; 3 Center for Vaccine Development and Global Health, University of Maryland School of Medicine, Baltimore, Maryland, United States

**Keywords:** adult immunisation, public health, vaccines, SARS-CoV-2, COVID-19, policy, joint reporting form

## Abstract

**Introduction:**

As SARS-CoV-2 disproportionately affects adults, the COVID-19 pandemic vaccine response will rely on adult immunisation infrastructures.

**Aim:**

To assess adult immunisation programmes in World Health Organization (WHO) Member States.

**Methods:**

We evaluated country reports from 2018 on adult immunisation programmes sent to WHO and UNICEF. We described existing programmes and used multivariable regression to identify independent factors associated with having them.

**Results:**

Of 194 WHO Member States, 120 (62%) reported having at least one adult immunisation programme. The Americas and Europe had the highest proportions of adult immunisation programmes, most commonly for hepatitis B and influenza vaccines (> 47% and > 91% of countries, respectively), while Africa and South-East Asia had the lowest proportions, with < 11% of countries reporting adult immunisation programmes for hepatitis B or influenza vaccines, and none for pneumococcal vaccines. In bivariate analyses, high or upper-middle country income, introduction of new or underused vaccines, having achieved paediatric immunisation coverage goals and meeting National Immunisation Technical Advisory Groups basic functional indicators were significantly associated (p < 0.001) with having an adult immunisation programme. In multivariable analyses, the most strongly associated factor was country income, with high- or upper-middle-income countries significantly more likely to report having an adult immunisation programme (adjusted odds ratio: 19.3; 95% confidence interval: 6.5–57.7).

**Discussion:**

Worldwide, 38% of countries lack adult immunisation programmes. COVID-19 vaccine deployment will require national systems for vaccine storage and handling, delivery and waste management to target adult risk groups. There is a need to strengthen immunisation systems to reach adults with COVID-19 vaccines.

## Introduction

From the outset of the coronavirus disease (COVID-19) pandemic, the global community has rapidly mobilised to develop vaccines against COVID-19. While the timely development, authorisation and manufacture of COVID-19 vaccines has been a public health imperative, rapid and global vaccine deployment is required to provide the greatest impact. The World Health Organization (WHO) has prioritised adult groups—including healthcare system workers, adults with chronic medical conditions and older adults—for COVID-19 vaccine receipt [1]. The global vaccine response will rely on existing immunisation infrastructures to reach these target groups [2]. It is therefore essential to understand what systems currently exist for adult immunisation and to identify any gaps, in order to effectively deploy COVID-19 vaccines.

While WHO recommends several vaccines for adults, global immunisation efforts have traditionally focused on young children. Since 1974, the Expanded Programme on Immunization (EPI) has been the major platform for vaccine delivery in low-income countries (LICs) and lower-middle-income countries (LMICs) [3]. Built on the global smallpox eradication infrastructure, EPI originally included four vaccines against six infectious diseases administered in the first year of life: bacille Calmette-Guerin, diphtheria-tetanus-pertussis, polio and measles vaccines [3]. Since then, EPI has been expanded to include several additional vaccines, with most targeting children in the first 2 years of life [4]. Exceptions include maternal tetanus toxoid immunisation in settings where routine paediatric tetanus immunisation is suboptimal and where maternal and neonatal tetanus have not been eliminated [5] and human papillomavirus vaccines (HPV) targeting girls aged 9 to 14 years [6]. WHO also recommends additional vaccines for adults in regions where certain diseases are endemic (including Japanese encephalitis and yellow fever vaccines), in high-risk populations (including influenza and rabies vaccines) and under special circumstances (such as in outbreak settings with Ebola virus disease or cholera vaccines) [4].

To gain an understanding of national immunisation programmes targeting adults globally, we reviewed adult immunisation data that countries reported to WHO and UNICEF in 2018 through the Joint Reporting Form on Immunisation (JRF). Our objectives were to identify countries with routine adult immunisation programmes and to determine factors associated with having these programmes, which could inform efforts to strengthen the vaccine response to the COVID-19 pandemic.

## Methods

### Primary data source

We collected data on national adult immunisation programmes from the JRF [7]. The JRF is a monitoring and evaluation tool that collects national administrative information regarding estimates of immunisation coverage, reported cases of vaccine-preventable diseases, immunisation schedules and campaigns, as well as indicators of immunisation system performance and financing [8]. The JRF is the only database of its kind that includes country-level data on immunisation programmes globally, making it the only dataset—to our knowledge—that could facilitate a global analysis of adult immunisation programmes. We chose five routine vaccines licensed for adult immunisation—hepatitis B vaccine (HepB), herpes zoster vaccine (HZV), influenza vaccine, pneumococcal conjugate vaccine (PCV) and pneumococcal polysaccharide vaccine (PPSV) (Table 1)—and accessed the 2018 JRF database on 12 February 2020 to collect country data on the presence of adult immunisation programmes for these vaccines. We chose not to review adult programmes for booster doses of routine childhood vaccines, vaccines that target regional endemic infections or those that are used primarily in outbreak settings.

**Table 1:** Summary of World Health Organization position papers for vaccine use in adults

Vaccine	Position statement year	Position for vaccine use in adults
Hepatitis B vaccine [25]	2017	The position paper states: *“WHO recommends hepatitis B vaccination of persons at high-risk of hepatitis B virus infection in [adults] and catch-up vaccination of unvaccinated cohorts if the necessary resources are available.”*
Herpes zoster vaccine [26]	2014	Countries that have an ageing population and elevated disease burden may choose to introduce HZV. Citing unknown burden of disease and insufficient data supporting HZV in most countries, the WHO does not offer any recommendation regarding the routine use of HZV.
Influenza vaccine [27]	2012	The position paper states: *“[I]ndividual national decisions on the use of influenza vaccines will be determined by national capacity and resources… For countries considering the initiation or expansion of programs for seasonal influenza vaccination, WHO recommends that pregnant women should have the highest priority. Additional risk groups to be considered for vaccination, in no particular order of priority, are children aged 6–59 months, [older adults], individuals with specific chronic medical conditions and healthcare workers.”*
Pneumococcal conjugate vaccine [4,28]	NA	WHO does not currently have recommendations on the use of PCV in individuals over 5 years of age, although addressing pneumococcal immunisation in adults is on the WHO Strategic Advisory Group of Experts on Immunization (SAGE) agenda for future deliberation.
Pneumococcal polysaccharide vaccine [29]	2008	The position paper states: *“Many industrialized countries recommend PPV23 immunization of their elderly and other high-risk groups. In resource-limited settings where there are many competing health priorities, the evidence does not support routine immunization of the elderly and high-risk populations with PPV23. Given the substantial effects of herd immunity in adult age groups following routine infant immunization with PCV7, a higher priority should be given to introducing and maintaining high coverage of infants with PCV7. Countries considering introducing PPV23 to [older adults] or other high-risk populations will need to develop strategies for reaching these target populations…The optimal timing, frequency and clinical effectiveness of additional doses of PPV23 are poorly defined, and national recommendations regarding revaccination vary. However, on the basis of the data on the duration of vaccine-induced protection, WHO suggests one single revaccination >5 years after a first vaccination.”*

HZV: herpes zoster vaccine; NA: not applicable; PCV: pneumococcal conjugate vaccine; PCV7: 7-valent pneumococcal polysaccharide-protein conjugate vaccine; PPV23: 23-valent non-conjugated pneumococcal polysaccharide vaccine; WHO: World Health Organization.

Summary of WHO position papers for vaccine use in adults are as at November 2020.

To assess whether countries are adopting global mandates for new vaccine introductions, we identified countries that had introduced rotavirus vaccine, HPV or birth dose HepB. Rotavirus vaccine and HPV are considered ‘new or underutilised’, and their introduction is prioritised by the Global Vaccine Action Plan (GVAP). Birth dose HepB is recommended by WHO, but has lower coverage than most other vaccines in the EPI schedule, so we also included it as an indicator of adoption of recommended immunisation programmes. To assess the general strength of routine immunisation, we classified countries according to whether they had achieved the GVAP goals of maternal and neonatal tetanus elimination (< 1 case of neonatal tetanus per 1,000 live births in every district of a country) [9] and whether they had achieved ≥ 95% national coverage of the third dose of diphtheria-tetanus-pertussis containing vaccine (DTP3), as per WHO/UNICEF estimates [10]. To evaluate a country’s capacity to make national decisions about immunisation programmes, we used WHO data indicating whether countries had functional National Immunization Technical Advisory Groups (NITAGs), meaning that they had achieved the following WHO-defined process indicators: (i) legislative or administrative basis for the advisory group, (ii) formal written terms of reference, (iii) at least five different areas of expertise represented among core members, (iv) at least one meeting per year, (v) circulation of the agenda and background documents at least one week before meetings and (vi) mandatory disclosure of any conflict of interest [9,11].

Questions in the 2018 JRF asked whether a country had adult programmes for HepB, HZV, influenza vaccine or PPSV. No specific question was included for adult PCV programmes, but there was a question about which groups were targeted for immunisation with PCV, with a free-text field for response. For PCV, when it was clear that an adult age group was targeted by a particular immunisation programme (such as when ‘adults’, ‘healthcare workers’ or ‘persons aged > 65 years old’ were indicated), we considered that the country had reported affirmatively that an adult programme was present. For countries that did not indicate the presence or absence of an adult immunisation programme in the JRF or that did not make clear whether an adult group was targeted for immunisation (such as when ‘high-risk groups’ was indicated), we considered that the country did not report having any adult immunisation programmes.

### Other data sources

We collected additional information to supplement the JRF data. To the extent possible, all covariate data were also from 2018, the year of the JRF dataset. We sought relevant country immunisation programme information from the websites of WHO Regional Offices, and we requested information from immunisation staff in each WHO Regional Office. Regional Office immunisation focal points are well-informed about routine immunisation programmes in their regions and are a part of the annual JRF data quality review. We therefore considered their concurrence to country reports to be an important quality check for this project. We also collected information about immunisation schedules for European Union/European Economic Area (EU/EEA) countries on the European Centre for Disease Prevention and Control (ECDC) website [12]. Next, we collected country economic information. As low-resource countries often rely on financial support from Gavi, the Vaccine Alliance, for new vaccine introduction, we identified all countries eligible for Gavi funding support in 2018 [13]. For per capita health expenditures, we used World Bank data for 2016, and we classified country income categories using the 2018 World Bank designations [14,15]. For population analyses, we used 2017 population estimates from the Institute for Health Metrics and Evaluation [16].

### Statistical analysis

As countries are not required to record the absence of immunisation programmes in the JRF for each of the vaccines we analysed, we could only record affirmative responses for some vaccines. For the analyses, we used the number of relevant WHO Member States as denominators, because we were unable to distinguish countries’ non-responses from negative responses. Further, PCV programme questions did not specify whether the programme was for adults. As many countries indicate the presence of paediatric pneumococcal immunisation programmes, we could not simply exclude countries from a denominator if they did not respond to a pneumococcal immunisation programme question. For statistical analyses, income group was defined as a categorical variable, the presence of adult immunisation programmes and health system/immunisation programme indicators were defined as dichotomous variables and per capita health expenditure was defined as a continuous variable.

We used descriptive statistics to describe countries with adult immunisation programmes. We conducted bivariate analyses to determine whether certain health system/immunisation programme characteristics were associated with the reported presence of an adult immunisation programme using chi-squared tests for categorical variables and Kruskal-Wallis tests for comparisons of medians. We calculated p values for trends using an extension of the Wilcoxon rank-sum test and adjusted odds ratios (aORs) using multiple logistic regression. The bivariate and multiple logistic regression analyses were performed on the complete global dataset and specifically for the WHO European Region. All statistical tests were two-sided, and p values < 0.05 were considered statistically significant. Analyses were conducted in Stata (version 15.1, StataCorp, College Station, Texas, United States (US)).

### Ethical statement

Human participants were not involved in this study, so institutional review board approval was not required.

## Results

### Data completeness

Among the 194 WHO Member States, 176 (90.7%) reported the presence or absence of a national adult immunisation programme in the JRF for at least one of the five assessed vaccines (HepB, HZV, influenza vaccine, PCV and PPSV). By Region, this ranged from 6 of 11 countries in South-East Asia to all 35 countries in the Americas. Regarding the presence of particular adult immunisation programmes, responses (either affirmative or negative) were available for 83 (42.8%) countries for HepB, 38 (19.6%) for HZV, 114 (58.8%) for influenza vaccine, 157 (80.9%) for PCV and 59 (30.4%) for PPSV. Otherwise, country JRF responses were missing (Supplementary Table S1).

Our review of ECDC’s country-level policy information for the EU/EEA identified 20 immunisation policies that were not reported in the 2018 JRF. Supporting information obtained from WHO Regional websites and Regional Office immunisation programme officers did not identify any new or conflicting data.

Percentages of countries with particular policies are presented as the number of countries that reported having (or were identified as having through additional efforts) particular immunisation programmes as numerators, with all WHO Member States (by WHO Region) as denominators.

### Immunisation programmes globally and by World Health Organization Region

Among the 194 WHO Member States, 71 (36.6%) countries reported having adult immunisation programmes for HepB, 17 (8.8%) for HZV, 114 (58.8%) for influenza vaccine, 16 (8.3%) for PCV and 35 (18.0%) for PPSV (Table 2). A total of 120 (61.9%) countries reported having any of the five assessed adult immunisation programmes, while three (1.5%) countries reported having programmes for each of the five vaccines studied.

**Table 2:** World Health Organization (WHO) Member States affirmatively reporting national adult immunisation programmes, by WHO Region and worldwide, 2018

WHO Region	n	Immunisation programme											
		HepB		HZV		Influenza vaccine		PCV		PPSV		Any of the assessed programmes	
		n	%	n	%	n	%	n	%	n	%	n	%
African	47	3	6.4	0	0.0	3	6.4	0	0.0	0	0.0	5	10.6
Americas	35	31	88.6	4	11.4	32	91.4	2	5.7	11	31.4	34	97.1
Eastern Mediterranean	21	7	33.3	1	4.8	13	61.9	1	4.8	2	9.5	14	66.7
European	53	25	47.2	10	18.9	50	94.3	12	22.6	18	34.0	50	94.3
South-East Asian	11	0	0.0	0	0.0	1	9.1	0	0.0	0	0.0	1	9.1
Western Pacific	27	5	18.5	2	7.4	15	55.6	1	3.7	4	14.8	16	59.3
Worldwide	194	71	36.6	17	8.8	114	58.8	16	8.3	35	18.0	120	61.9

HepB: hepatitis B vaccine; HZV: herpes zoster vaccine; PCV: pneumococcal conjugate vaccine; PPSV: pneumococcal polysaccharide vaccine; WHO: World Health Organization.

**Figure 1:**
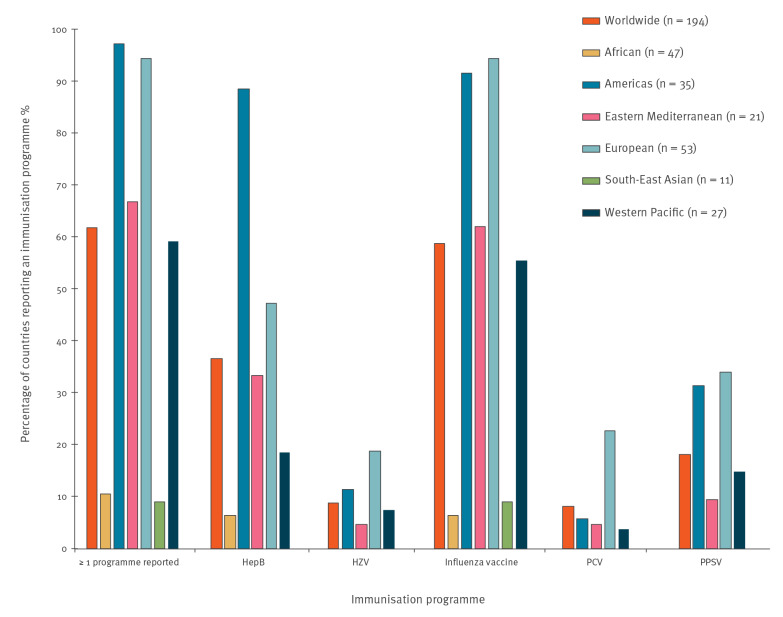
Reported adult immunisation programmes, by World Health Organization Region, 2018

Adult immunisation programme data by WHO Region are shown in Figure 1 and Table 2. In the African Region (47 countries), five countries reported at least one adult immunisation programme. African countries had programmes for HepB (three countries) and influenza vaccine (three countries). No African countries reported adult immunisation programmes for HZV, PCV or PPSV.

HepB: hepatitis B vaccine; HZV: herpes zoster vaccine; PCV: pneumococcal conjugate vaccine; PPSV: pneumococcal polysaccharide vaccine.

Source: 2018 World Health Organization/UNICEF Joint Reporting Form [7].

In the Region of the Americas (35 countries), 34 countries reported at least one adult immunisation programme. Countries in the Americas had programmes for PCV (two countries), HZV (four countries), PPSV (11 countries), HepB (31 countries) and influenza vaccine (32 countries).

In the Eastern Mediterranean Region (21 countries), 14 countries reported at least one adult immunisation programme. Eastern Mediterranean countries had programmes for HZV and PCV (one country each), PPSV (two countries), HepB (seven countries) and influenza vaccine (13 countries).

In the European Region (53 countries), 50 countries reported at least one adult immunisation programme. European countries had programmes for HZV (10 countries), PCV (12 countries), PPSV (18 countries), HepB (25 countries) and influenza vaccine (50 countries).

In the South-East Asian Region (11 countries), one country reported at least one adult immunisation programme (for influenza vaccine). No South-East Asian countries reported programmes for HepB, HZV, PCV or PPSV.

In the Western Pacific Region (27 countries), 16 countries reported at least one adult immunisation programme. Western Pacific countries had programmes for PCV (one country), HZV (two countries), PPSV (four countries), HepB (five countries) and influenza vaccine (15 countries).

We compared the populations of countries with and without adult immunisation programmes. Globally, 38.7% of the world’s population lives in a country reporting at least one adult immunisation programme (Table 3). The percentage of the global population living in countries reporting specific adult immunisation programmes is as follows: 13.3% for HepB, 9.0% for HZV, 36.7% for influenza vaccine, 30.9% for PCV and 19.1% for PPSV. The percentage of persons living in countries reporting at least one adult immunisation programme, by WHO Region, is: 13.9% for the African Region, 98.8% for the Region of the Americas, 51.2% for the Eastern Mediterranean Region, 97.2% for the European Region, 3.5% for the South-East Asian Region and 24.1% for the Western Pacific Region.

**Table 3:** Population in countries reporting national adult immunisation programmes, by World Health Organization Region and worldwide, 2018

WHO Region	Total population (millions)	Population (by immunisation programme reported)											
		HepB		HZV		Influenza vaccine		PCV		PPSV		Any of the assessed vaccines	
		n (millions)	%	n (millions)	%	n (millions)	%	n (millions)	%	n (millions)	%	n (millions)	%
African	1,049.6	50.7	4.8	0	0.0	96.7	9.2	0	0.0	0	0.0	146.1	13.9
Americas	1,002.9	686.2	68.4	412.0	41.1	990.2	98.7	328.3	32.7	640.4	63.9	991.0	98.8
Eastern Mediterranean	706.1	157.9	22.4	9.7	1.4	265.3	37.6	93.4	13.2	18.2	2.6	361.8	51.2
European	932.5	79.6	8.5	231.7	24.8	906.6	97.2	160.5	17.2	476.7	51.1	906.6	97.2
South-East Asian	1,999.0	0	0.0	0	0.0	70.6	3.5	0	0.0	0	0.0	70.6	3.5
Western Pacific	1,896.1	36.0	1.9	28.4	1.5	456.0	24.0	4.4	0.2	314.0	16.6	456.9	24.1
Worldwide	7,586.3	1,010.4	13.3	681.8	9.0	2,785.4	36.7	586.6	7.7	1,449.4	19.1	2,933.1	38.7

HepB: hepatitis B vaccine; HZV: herpes zoster vaccine; PCV: pneumococcal conjugate vaccine; PPSV: pneumococcal polysaccharide vaccine; WHO: World Health Organization.

Population data are from 2017 [16].

### Economic indicators and characteristics of national immunisation systems

**Figure 2:**
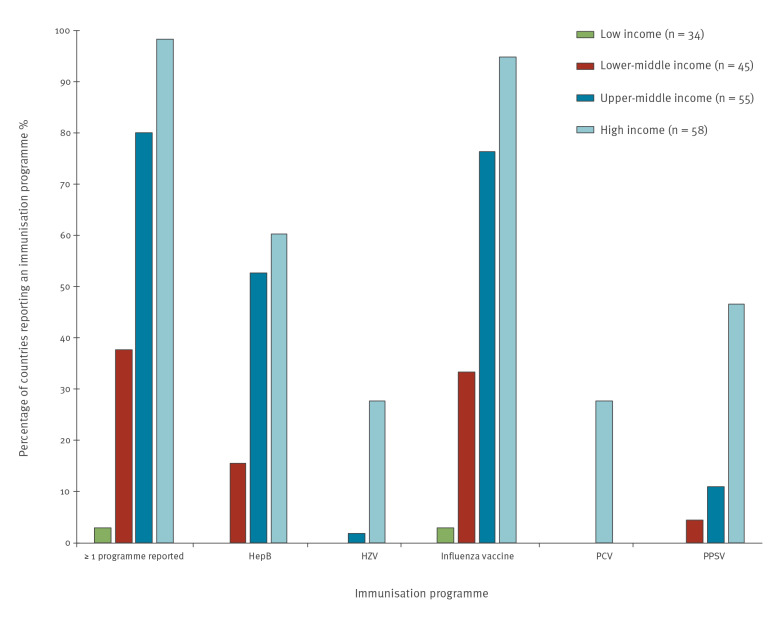
Reported adult immunisation programmes, by World Bank income categories, 2018

In bivariate analyses, economic indicators were highly associated with having at least one adult immunisation programme (Figure 2 and Table 4). Countries classified by the World Bank as high or upper-middle income were more likely to have an adult immunisation programme than those classified as low or lower-middle income (p < 0.001). Countries with an adult immunisation programme also had higher median per capita health expenditures compared with those without any programmes (p < 0.001), with the same being true for the individual adult immunisation programmes (Supplementary Tables S2 and S3).

HepB: hepatitis B vaccine; HZV: herpes zoster vaccine; PCV: pneumococcal conjugate vaccine; PPSV: pneumococcal polysaccharide vaccine.

World Bank income category data are from 2016 [14].

Source: 2018 World Health Organization/UNICEF Joint Reporting Form [30].

**Table 4:** Characteristics of World Health Organization Member States, by whether they reported adult immunisation programmes, 2018

Characteristics	Reported at least one adult immunisation programme^a^						
	Yes n = 120			No n = 74			p value^b^
	n		%	n		%	
**Median per capita health expenditure in Int$ (IQR)^c^**	**854 (396–2,215)**			**58 (22–153)**			**< 0.001^d^**
**World Bank income group^e^**							
Low income	1	0.8		33	45.2		< 0.001^f^
Lower-middle income	18	15.1		28	38.4		
Upper-middle income	45	37.8		11	15.1		
High income	55	46.2		1	1.4		
**Eligible for Gavi, the Vaccine Alliance funding [** 19 **]**							
Yes	1	0.8		47	63.5		< 0.001
No	119	99.2		27	36.5		
**Introduced hepatitis B vaccine birth dose**							
Yes	94	78.3		34	45.9		< 0.001
No	26	21.7		40	54.1		
**Introduced human papilloma virus vaccine**							
Yes	76	63.3		14	18.9		< 0.001
No	44	36.7		60	81.1		
**Introduced rotavirus vaccine**							
Yes	60	50.0		41	55.4		0.46
No	60	50.0		33	44.6		
**Has a functional NITAG**							
Yes	82	68.3		32	43.2		< 0.01
No	38	31.7		42	56.8		
**Eliminated maternal and neonatal tetanus**							
Yes	120	100.0		60	81.1		< 0.001
No	0	0.0		14	18.9		
**Third dose diphtheria-tetanus-pertussis coverage is ≥ 95% nationally**							
Yes	66	55.0		18	24.3		< 0.001
No	54	45.0		56	75.7		

IQR: interquartile range; NITAG: National Immunization Technical Advisory Group.

^a^ Reported an adult immunisation programme for any of the following: hepatitis B vaccine, herpes zoster vaccine, influenza vaccine, pneumococcal conjugate vaccine, pneumococcal polysaccharide vaccine.

^b^ Chi-squared test, unless indicated otherwise.

^c^ Excluding 11 countries with missing healthcare expenditure data.

^d^ Kruskal-Wallis test for difference in medians.

^e^ Denominator = 192 countries; 119 with an adult immunisation programme and 73 without an adult immunisation programme. Niue and The Cook Islands, not World Bank member countries, are excluded from the income categories. All data are from 2018 except per capita health expenditures, which are from 2016, the most recent year for which such data were available from the World Bank. Per capita health expenditure data are calculated by the World Bank in International Dollars (Int$), which—by definition—would buy a comparable amount of goods and services in the cited country as the USD would buy in the United States.

^f^ P value for trend.

Certain characteristics of national immunisation systems were associated with reporting an adult immunisation programme (Table 4). Compared with countries that did not report any adult immunisation programmes, countries that reported at least one adult programme were more likely to have included the HepB birth dose in their routine immunisation schedule (94/120, 78.3% vs 34/74, 45.9%; p < 0.001), to have eliminated maternal and neonatal tetanus (120/120, 100% vs 60/74, 81.1%; p < 0.001) and to have achieved national DTP3 coverage ≥ 95% (66/120, 55.0% vs 18/74, 24.3%; p < 0.001). Having introduced HPV was also associated with having at least one adult programme (76/120, 46.3% vs 14/74, 18.9%; p < 0.001), as well as with having each of the five individual adult programmes (Supplementary Table S4). The introduction of rotavirus vaccine was not associated with the presence of an adult immunisation programme (Table 3). Finally, countries reporting an adult immunisation programme were more likely to have a functional NITAG than those not reporting an adult programme (82/120, 68.3% vs 32/74, 43.2%; p < 0.001).

In a multiple logistic regression model that included each of the binary national immunisation system indicators (≥ 95% DTP3 coverage, presence of a functional NITAG and introduction of a birth dose of HepB, rotavirus vaccine and HPV) and was adjusted for income category (LIC/LMIC vs upper-middle-income country (UMIC)/high-income country (HIC)), the presence of a functional NITAG (aOR: 7.1; 95% confidence interval (CI): 2.5–20.7), the introduction of the HepB birth dose (aOR: 3.6; 95% CI: 1.4–10.4) and the introduction of HPV (aOR: 3.9; 95% CI: 4.3–10.4) remained significantly associated with reporting an adult immunisation programme (Supplementary Table S5).

Country income group was the factor most strongly associated with whether a country reported an adult immunisation programme (aOR: 21.9; 95% CI: 7.3–65.3) for HIC/UMIC countries compared with LIC/LMIC countries. In a model limited to WHO Member States classified as LICs or LMICs, the aOR for having at least one adult immunisation programme was 12.0 (95% CI: 2.3–63.4) for countries with a functional NITAG compared with those without one, and 5.9 (95% CI: 1.5–4.2) for countries that had introduced a birth dose of HepB in their EPI schedule, compared with those that had not. Multiple logistic regression models for the individual adult immunisation programmes showed similar trends (Supplementary Table S6).

### World Health Organization European Region

To gain further insight into adult immunisation programmes in the WHO European Region, we conducted bivariate and multiple logistic regression analyses restricted to the 53 countries in this Region. Of these countries, three had no adult immunisation programmes. In bivariate analyses, countries with at least one adult immunisation programme had greater median per capita health expenditures (calculated by the World Bank using International Dollars (Int$), which—by definition—would buy a comparable amount of goods and services in the cited country as the USD would buy in the United States) compared with those without any adult programmes (Int$ 1,616 vs Int$ 93; p < 0.01) and were less likely to be eligible for Gavi funding (0/50, 0% vs 2/3, 66.7%; < 0.001) (Supplementary Table S7). Having introduced HPV was the only characteristic associated with having one or more adult immunisation programme (35/50, 70.0% vs 0/3, 33.3%; p = 0.01).

In multivariable analyses adjusting for rotavirus introduction, having a functional NITAG, having DTP3 coverage ≥ 95% and being a HIC or UMIC, the only characteristic significantly associated with having an adult immunisation programme was being a HIC or UMIC (45/50, 90% vs 1/3, 33.3%;, aOR: 37.3; 95% CI: 1.5–962.2) (Supplementary Table S7). In multivariable analyses of the individual adult immunisation programmes, having introduced HPV was independently associated with having an adult HepB programme (20/25, 80% vs 15/28, 53.6%; aOR: 5.6; 95% CI: 1.3–24.2) and with having an adult PPSV programme (17/18, 94.4% vs 18/35, 51.4%; aOR: 11.7; 95% CI: 1.2–112.4). In the European Region, being a HIC or UMIC, rather than a LMIC (as there were no European Region countries categorised as a LIC), was independently associated with having an adult influenza immunisation programme (45/50, 90% vs 1/3, 33.3%; aOR: 37.3, 95% CI: 1.4–962.2) (Supplementary Table S8).

## Discussion

In our review of JRF data from 2018, 61.9% of WHO Member States reported having at least one adult immunisation programme, including countries from all WHO Regions and income categories. The most common adult immunisation programme was for influenza vaccines, reported by 58.8% of countries, while adult immunisation programmes for other vaccines were much less common. The number of adult influenza immunisation programmes has increased since the 2014 JRF, when 46% of countries reported programmes for adults with chronic disease and 45% reported programmes for older adults [8]. In that analysis, countries reporting influenza immunisation programmes were wealthier and more likely to have functional NITAGs, to have introduced new or under-used vaccines and to have stronger immunisation systems.

Our study documents and quantifies major limitations to routine adult immunisation programmes in a substantial proportion of countries globally and indicates that the inequities previously reported for influenza immunisation programmes in 2014 are also true for adult immunisation programmes in general [8]. The fact that 38.1% of countries worldwide lack functional systems to deliver routine adult immunisation has major implications for COVID-19 vaccine deployment.

WHO immunisation position papers are permissive towards national immunisation programmes for the adult vaccines we assessed, indicating that countries may choose to target adults for immunisation, but no WHO mandates for such programmes are in place. However, at a 2017 WHO meeting of experts it was acknowledged that there were data gaps hindering adult immunisation programme policy-making globally, particularly in LICs and LMICs [2]. Investment in studies to better measure disease burden and the potential impact of adult immunisation programmes are needed to inform the value proposition of adult immunisation in low-resource settings. Further, while global vaccine policy has often been driven by estimates of mortality prevention or cost-effectiveness, the full impact of adult vaccine-preventable diseases may be better understood by different metrics [2]. The impact on a community when older adults lose independence or functional capacity after a vaccine-preventable illness is difficult to assess, and the value of missed unpaid work in the home or as a child caregiver is seldom measured in economic analyses [17]. The presence of favourable global policies for adult immunisation is not sufficient to advance immunisation implementation globally if impact estimates undervalue older adults’ lives or their contributions to society.

While we do not necessarily advocate for the expansion of routine adult immunisation services into LICs and LMICs, such programmes can provide public health value. In addition to the direct impact of routine immunisation, there are further benefits of strong adult immunisation programmes. For example, they can provide platforms for the delivery of other preventive interventions and strengthen primary care [18], and they can enhance public health responses during public health emergencies requiring immunisation campaigns, such as the current COVID-19 pandemic. In 2009, having a seasonal influenza immunisation programme was significantly associated with the deployment of pandemic influenza vaccines when they became available [19]. This was attributed to infrastructure preparedness as well as individual attitudes regarding immunisation, both of which are enhanced when a country has a functional adult influenza immunisation programme [19]. Finally, strengthening countries’ capacities to provide immunisation services across the life course should occur concurrently with the development of new vaccines targeting adult age groups, so that delivery systems will be in place once new vaccines become available.

A critical component of strong national immunisation systems is the presence of a NITAG, and we found an association between having at least one adult immunisation programme and having a functional NITAG. Within an individual country, the NITAG plays a critical role in evaluating vaccines, interpreting available safety and efficacy data and applying relevant data to policy recommendations most appropriate for a given country. NITAGs may consider targeting healthcare workers for influenza immunisation as the most logical first step to advancing immunisation in LICs and LMICs, as there are existing systems to transport, store and deliver vaccines within healthcare systems [20]; the total population of healthcare workers is small compared with other influenza risk groups; and sensitising healthcare workers to the benefits of immunisation has effects on their advocacy of vaccines to patients [21]. A healthy medical workforce is critical to ensuring that essential healthcare services continue during disease epidemics.

This study should be interpreted in the context of its strengths and limitations. To our knowledge, the JRF is the only global source of national immunisation data and is therefore the only dataset that could facilitate a quantification of adult immunisation programmes globally. The survey is a routine public health instrument that is completed annually by each country’s Ministry of Health, with extensive quality checks at the WHO Regional and global levels. It is an important platform for monitoring adult vaccine policy development over time; however, the JRF relies on national self-reporting, and there may be errors introduced at the country level that would be difficult to identify or correct. These data capture only what is recommended as public policy, though they may not describe vaccine use in the private sector. Further, some questions in the JRF, particularly for PCV, require countries to add a free-text response to indicate a programme’s target groups, making it difficult to confirm whether an adult immunisation program is present. WHO has moved to a web-based application to collect the 2019 JRF data, which will provide opportunities to improve the questionnaire and decrease potential reporting bias. We believe that engaging WHO Regional Office immunisation focal points as a data quality check was a strength to this study, as they are highly informed about the routine immunisation programmes within their regions and are likely to know about any adult immunisation programmes, particularly in LICs and LMICs.

Finally, the presence of a national adult immunisation programme does not necessarily indicate substantial vaccine use. In a 2015 review of global influenza vaccine distribution, the International Federation of Pharmaceutical Manufacturers and Associations estimated that ca 95% of global influenza vaccine distribution occurs in the Americas, Europe and the Western Pacific [22], despite 17 countries reporting adult influenza immunisation programmes outside of these WHO Regions. While the JRF does collect information on influenza immunisation coverage of older adults and persons with chronic medical conditions, the data are substantially incomplete, making analyses of global influenza vaccine use in these risk groups infeasible [8]. Our analysis determined whether routine national programmes were in place to immunise adults, but it could not ascertain whether the systems are sufficient for COVID-19 immunisation campaigns. WHO planned for an initial tranche of vaccines to cover 3% of national populations [23] and, as at April 2021, COVID-19 vaccine coverage remains low in many LICs and LMICs [24]. While the real percentage may differ from country to country based on advanced market purchase agreements, limited supplies of COVID-19 vaccines to date may limit stress on some of the immunisation systems deploying them, globally.

### Conclusions

Nearly 40% of countries worldwide have no immunisation infrastructure to provide adult immunisation and nearly 60% of the world’s population lives in countries without routine adult immunisation programmes. COVID-19 vaccines are being deployed in many countries that do not have routine adult immunisation infrastructure. Social mobilisation and outreach, as well as vaccine storage, handling, delivery and waste management for adult immunisation do not exist in much of the world and will have to be developed to support the COVID-19 pandemic response. Our study suggests that the global response to the COVID-19 pandemic should address disparities in adult immunisation systems, in order to maximise the impact and equity of COVID-19 vaccine deployment.

## Supplementary Data

953000 Supplementary Material
