# Supplementary material for: National routine adult immunisation programmes among World Health Organization Member States: an assessment of health systems to deploy COVID-19 vaccines
Source: Euro Surveill. 2021 Apr 29;26(17):2001195. doi: 10.2807/1560-7917.ES.2021.26.17.2001195 (PMC8086245; doi:10.2807/1560-7917.ES.2021.26.17.2001195)
Supplement: Supplementary Material [file 2001195_SupplementaryMaterial.pdf]

**SUPPLEMENTARY MATERIAL**

This supplementary material is hosted by *Eurosurveillance* as supporting information alongside the article "National routine adult immunisation programmes among World Health Organization member states: an assessment of health systems to deploy SARS-CoV-2 vaccines" on behalf of the authors who remain responsible for the accuracy and appropriateness of the content. The same standards for ethics, copyright, attributions and permissions as for the article apply. Supplements are not edited by *Eurosurveillance* and the journal is not responsible for the maintenance of any links or email addresses provided therein.

**Title:** National routine adult immunisation programmes among World Health Organization member states: an assessment of health systems to deploy SARS-CoV-2 vaccines

**Authors:** Sarah R Williams,[1,2] Amanda J Driscoll,[2,3] Hanna M LeBuhn,[3] Wilbur H Chen,[3] Kathleen M Neuzil,[3] Justin R Ortiz[3]

1. Division of Pulmonary and Critical Care Medicine, University of Maryland School of Medicine, Baltimore, Maryland, United States
2. These authors contributed equally to this manuscript
3. Center for Vaccine Development and Global Health, University of Maryland School of Medicine, Baltimore, Maryland, United States

**Table S1. Countries responding to specific JRF question regarding presence of an adult immunization programme, by vaccine**

| WHO Region | Hepatitis B Vaccine | | Herpes Zoster Vaccine | | Influenza Vaccine | | Pneumococcal Conjugate Vaccine | | Pneumococcal Polysaccharide Vaccine | | Any of the Assessed Vaccines | |
|---|---|---|---|---|---|---|---|---|---|---|---|---|
| | n | % | n | % | n | % | n | % | n | % | n | % |
| African (n=47) | 3 | 6.4 | 0 | 0.0 | 3 | 6.4 | 41 | 87.2 | 0 | 0.0 | 41 | 87.2 |
| Americas (n=35) | 31 | 88.6 | 4 | 11.4 | 32 | 91.4 | 28 | 80.0 | 16 | 45.7 | 35 | 100.0 |
| Eastern Mediterranean (n=21) | 7 | 33.3 | 1 | 4.8 | 13 | 61.9 | 16 | 76.2 | 4 | 19.1 | 20 | 95.2 |
| European (n=53) | 37 | 69.8 | 31 | 58.5 | 50 | 94.3 | 48 | 90.6 | 34 | 64.2 | 52 | 98.1 |
| South-East Asian (n=11) | 0 | 0.0 | 0 | 0.0 | 1 | 9.1 | 5 | 45.5 | 0 | 0.0 | 6 | 54.6 |
| Western Pacific (n=27) | 5 | 18.5 | 2 | 7.4 | 15 | 55.6 | 19 | 70.4 | 5 | 18.5 | 22 | 81.5 |
| Worldwide (n=194) | 83 | 42.8 | 38 | 19.6 | 114 | 58.8 | 157 | 80.9 | 59 | 30.4 | 176 | 90.7 |

**Table S2. Economic characteristics of WHO member states in 2018, by reported national adult immunization programmes**

| | Hepatitis B Vaccine Programme | | | | | Herpes Zoster Vaccine Programme | | | | | Influenza Vaccine Programme | | | | | Pneumococcal Conjugate Vaccine Programme | | | | | Pneumococcal Polysaccharide Vaccine Programme | | | | |
|---|---|---|---|---|---|---|---|---|---|---|---|---|---|---|---|---|---|---|---|---|---|---|---|---|---|
| WB Income group[2] | Yes (n=72) | | No (n=121) | | p-value[1] | Yes (n=17) | | No (n=175) | | p-value | Yes (n=113) | | No (n=79) | | p-value | Yes (n=16) | | No (n=176) | | p-value | Yes (n=35) | | No (n=157) | | p-value |
| | n | % | n | % | | n | % | n | % | | n | % | n | % | | n | % | n | % | | n | % | n | % | |
| Low income (n=34) | 0 | 0.0 | 34 | 28.1 | <0.001 | 0 | 0.0 | 34 | 19.4 | <0.001 | 1 | 0.9 | 33 | 41.8 | <0.001 | 0 | 0.0 | 34 | 19.3 | <0.001 | 0 | 0.0 | 34 | 21.7 | <0.001 |
| Lower-middle income (n=46) | 7 | 9.9 | 39 | 32.1 | | 0 | 0.0 | 46 | 26.3 | | 15 | 13.3 | 31 | 39.2 | | 0 | 0.0 | 46 | 26.1 | | 2 | 5.7 | 44 | 28.0 | |
| Upper middle income (n=56) | 29 | 40.9 | 27 | 22.3 | | 1 | 5.9 | 55 | 31.4 | | 42 | 37.2 | 14 | 17.7 | | 0 | 0.0 | 56 | 31.8 | | 6 | 17.1 | 50 | 31.9 | |
| High income (n=56) | 35 | 49.3 | 21 | 17.4 | | 16 | 94.1 | 40 | 22.9 | | 55 | 48.7 | 1 | 1.3 | | 16 | 100.0 | 40 | 22.7 | | 27 | 77.1 | 29 | 18.5 | |
| Gavi eligible | Yes (n=72) | | No (n=123) | | p-value[1] | Yes (n=17) | | No (n=177) | | p-value | Yes (n=114) | | No (n=80) | | p-value | Yes (n=16) | | No (n=178) | | p-value | Yes (n=35) | | No (n=159) | | p-value |
| | n | % | n | % | | n | % | n | % | | n | % | n | % | | n | % | n | % | | n | % | n | % | |
| Yes (n=48) | 1 | 1.4 | 47 | 38.2 | <0.001 | 0 | 0.0 | 48 | 27.1 | 0.01 | 0 | 0.0 | 48 | 60.0 | <0.001 | 0 | 0.0 | 48 | 27.0 | 0.02 | 0 | 0.0 | 48 | 31.8 | <0.001 |
| No (n=146) | 70 | 98.6 | 76 | 61.8 | | 17 | 100.0 | 129 | 72.9 | | 114 | 100.0 | 32 | 40.0 | | 16 | 100.0 | 130 | 73.0 | | 43 | 100.0 | 103 | 68.2 | |

1. p-value for trend (World Bank income group comparisons)
2. Niue and The Cook Islands, not World Bank member countries, are excluded from the income categories
3. Reference category
4. Chi-square test (Gavi eligibility comparisons)

**Table S3. Median per capita health care expenditures of WHO member states in 2018, by reported national adult immunization Programme**

| Adult Vaccination Programme | Has programme | Statistic | Median per capita health care expenditure (Int$)[1] |
|---|---|---|---|
| Hepatitis B Vaccine Programme | Yes (n=68) | Median (IQR) | 884 (412, 2118) |
| | No (n=116) | Median (IQR) | 195 (45, 732) |
| | | p-value[2] | <0.001 |
| Herpes Zoster Vaccine Programme | Yes (n=17) | Median (IQR) | 2,882 (1,845, 3,465) |
| | No (n=167) | Median (IQR) | 314 (62, 900) |
| | | p-value | <0.001 |
| Influenza Vaccine Programme | Yes (n=109) | Median (IQR) | 933 (482, 2,226) |
| | No (n=75) | Median (IQR) | 59 (25, 196) |
| | | p-value | <0.001 |
| Pneumococcal Conjugate Vaccine Programme | Yes (n=16) | Median (IQR) | 2,221 (1335, 3510) |
| | No (n=168) | Median (IQR) | 319 (63, 884) |
| | | p-value | <0.001 |
| Pneumococcal Polysaccharide Vaccine Programme | Yes (n=43) | Median (IQR) | 1,676 (930, 3,331) |
| | No (n=151) | Median (IQR) | 208 (52, 564) |
| | | p-value | <0.001 |

[1]Economic data are from 2016, the most recent year for which such data were available from the World Bank. Data are calculated by the World Bank in International Dollars (Int$), which—by definition—would buy a comparable amount of goods and services in the cited country as the USD would buy in the United States. Excluding 10 countries with missing health care expenditure data
[2]Kruskal-Wallis test for difference in medians

**Table S4. Immunization programme characteristics of WHO member states in 2018, by reported national adult immunization programmes**

| | | Hepatitis B Vaccine Programme | | | | | Herpes Zoster Vaccine Programme | | | | | Influenza Vaccine Programme | | | | | Pneumococcal Conjugate Vaccine Programme | | | | | Pneumococcal Polysaccharide Vaccine Programme | | | | |
|---|---|---|---|---|---|---|---|---|---|---|---|---|---|---|---|---|---|---|---|---|---|---|---|---|---|---|
| | n | Yes | | No | | p-value | Yes | | No | | p-value | Yes | | No | | p-value | Yes | | No | | p-value | Yes | | No | | p-value |
| | | n | % | n | % | | n | % | n | % | | n | % | n | % | | n | % | n | % | | n | % | n | % | |
| Introduced Hepatitis B birth dose | | | | | | | | | | | | | | | | | | | | | | | | | | |
| Yes | 128 | 53 | 74.6 | 75 | 61.0 | 0.05 | 14 | 82.4 | 114 | 64.4 | 0.14 | 91 | 79.8 | 37 | 46.3 | <0.001 | 12 | 75.0 | 116 | 62.5 | 0.43 | 27 | 77.1 | 101 | 78.9 | 0.12 |
| No | 66 | 18 | 25.4 | 48 | 39.0 | | 3 | 17.6 | 63 | 35.6 | | 23 | 20.2 | 43 | 53.8 | | 4 | 25.0 | 62 | 34.8 | | 8 | 22.6 | 58 | 87.9 | |
| Introduced HPV | | | | | | | | | | | | | | | | | | | | | | | | | | |
| Yes | 90 | 50 | 70.4 | 40 | 32.5 | <0.001 | 17 | 100.0 | 73 | 41.2 | <0.001 | 73 | 64.0 | 17 | 21.3 | <0.001 | 13 | 81.3 | 77 | 43.3 | <0.01 | 29 | 82.9 | 61 | 38.4 | <0.001 |
| No | 104 | 21 | 29.6 | 83 | 67.5 | | 0 | 0.0 | 104 | 58.8 | | 41 | 36.0 | 63 | 78.8 | | 3 | 18.8 | 101 | 56.7 | | 6 | 17.1 | 98 | 61.6 | |
| Introduced Rotavirus vaccine | | | | | | | | | | | | | | | | | | | | | | | | | | |
| Yes | 101 | 40 | 56.3 | 61 | 49.6 | 0.37 | 13 | 76.5 | 88 | 49.7 | 0.04 | 56 | 49.1 | 45 | 56.3 | 0.33 | 10 | 62.5 | 91 | 51.1 | 0.38 | 23 | 65.7 | 78 | 49.1 | 0.07 |
| No | 93 | 31 | 43.7 | 62 | 50.4 | | 4 | 23.5 | 89 | 50.3 | | 58 | 50.9 | 35 | 43.8 | | 6 | 37.5 | 87 | 48.9 | | 12 | 37.1 | 81 | 50.9 | |
| Functioning NITAG[2] | | | | | | | | | | | | | | | | | | | | | | | | | | |
| Yes | 114 | 47 | 66.2 | 67 | 54.5 | 0.11 | 14 | 82.4 | 100 | 56.5 | 0.04 | 78 | 68.4 | 36 | 45.0 | <0.01 | 13 | 81.3 | 101 | 56.7 | 0.06 | 23 | 65.7 | 91 | 57.2 | 0.36 |
| No | 80 | 24 | 33.8 | 56 | 45.5 | | 3 | 17.6 | 77 | 43.5 | | 36 | 31.6 | 44 | 55.0 | | 3 | 18.8 | 77 | 43.3 | | 12 | 37.1 | 68 | 42.8 | |
| Eliminated maternal and neonatal tetanus | | | | | | | | | | | | | | | | | | | | | | | | | | |
| Yes | 180 | 71 | 100.0 | 109 | 88.6 | <0.01 | 17 | 100.0 | 163 | 92.1 | 0.23 | 114 | 100.0 | 66 | 82.5 | <0.001 | 16 | 100.0 | 164 | 92.1 | 0.24 | 35 | 100.0 | 145 | 91.2 | 0.07 |
| No | 14 | 0 | 0.0 | 14 | 11.4 | | 0 | 0.0 | 14 | 7.9 | | 0 | 0.0 | 14 | 17.5 | | 0 | 0.0 | 14 | 7.9 | | 0 | 0.0 | 14 | 8.8 | |
| DTP coverage ≥95% nationally | | | | | | | | | | | | | | | | | | | | | | | | | | |
| Yes | 84 | 39 | 54.9 | 45 | 36.6 | 0.01 | 7 | 41.2 | 77 | 43.5 | 0.85 | 62 | 54.4 | 22 | 27.5 | <0.001 | 10 | 62.5 | 74 | 41.6 | <0.01 | 18 | 51.4 | 66 | 41.5 | 0.28 |
| No | 110 | 32 | 45.1 | 78 | 63.4 | | 10 | 58.8 | 100 | 56.5 | | 52 | 45.6 | 58 | 72.5 | | 6 | 37.5 | 104 | 58.4 | | 17 | 48.6 | 93 | 58.5 | |

HPV= human papillomavirus vaccine; DTP= diphtheria, tetanus and pertussis containing vaccine

1. Chi-square test
2. National Immunization Technical Advisory Group; limited to n=134 countries for which this information was available

**Table S5. Analysis of immunization programme characteristics associated with the presence of one or more adult vaccination programmes**

| | Any adult vaccination programme | | |
|---|---|---|---|
| | Yes (n=120) | | |
| | n | % | aOR[1] (95% CI) |
| Introduced Hepatitis B birth dose | | | |
| Yes | 94 | 78.3 | 3.26 (1.2, 9.0) |
| No | 26 | 21.7 | |
| Introduced HPV | | | |
| Yes | 76 | 63.3 | 3.48 (1.3, 9.4) |
| No | 44 | 36.7 | |
| Introduced Rotavirus vaccine | | | |
| Yes | 60 | 50.0 | 1.13 (0.4, 2.9) |
| No | 60 | 50.0 | |
| Functional NITAG[2] | | | |
| Yes | 82 | 68.3 | 6.74 (2.3, 19.7) |
| No | 38 | 31.7 | |
| Eliminated maternal and neonatal tetanus | | | |
| Yes | 120 | 100.0 | n/a |
| No | 0 | 0.0 | |
| DTP coverage ≥95% nationally | | | |
| Yes | 66 | 55.0 | 1.57 (0.6, 3.9) |
| No | 54 | 45.0 | |
| HIC or UMIC[2] | | | |
| Yes | 100 | 84.0 | 19.28 (6.5, 57.7) |
| No | 19 | 16.0 | |

1. Adjusted for all other characteristics in the table
2. Two countries without world bank income classifications excluded from denominator

**Table S6. Analysis of immunization programme characteristics associated with the presence of individual adult immunisation programmes**

| | Adult HepB programme | | | Adult HZV programme | | | Adult influenza vaccination programme | | | Adult PCV programme | | | Adult PPSV programme | | |
|---|---|---|---|---|---|---|---|---|---|---|---|---|---|---|---|
| | Yes (n=71) | | | Yes (n=17) | | | Yes (n=114) | | | Yes (n=16) | | | Yes (n=35) | | |
| | n | % | aOR (95% CI) | n | % | aOR (95% CI) | n | % | aOR[1] (95% CI) | n | % | aOR (95% CI) | n | % | aOR (95% CI) |
| Introduced Hepatitis B birth dose | | | | | | | | | | | | | | | |
| Yes | 53 | 74.7 | 1.0 (0.4, 2.4) | 14 | 82.4 | 3.8 (1.0, 14.6) | 91 | 79.8 | 4.1 (1.5, 10.9) | 12 | 75.0 | 1.5 (0.5, 5.2) | 27 | 77.1 | 1.3 (0.5, 3.4) |
| No | 18 | 25.4 | | 3 | 17.7 | | 23 | 20.2 | | 4 | 25.0 | | 8 | 22.9 | |
| Introduced HPV | | | | | | | | | | | | | | | |
| Yes | 50 | 70.4 | 1.9 (0.9, 4.2) | 17 | 100.0 | n/a | 73 | 64.0 | 3.8 (1.4, 9.9) | 13 | 81.3 | 4.7 (1.3, 17.5) | 29 | 82.9 | 3.3 (1.2, 9.6) |
| No | 21 | 29.6 | | 0 | 0.0 | | 41 | 36.0 | | 3 | 18.8 | | 6 | 17.1 | |
| Introduced Rotavirus vaccine | | | | | | | | | | | | | | | |
| Yes | 40 | 56.3 | 1.9 (0.9, 3.9) | 13 | 76.5 | 4.2 (1.2, 14.3) | 56 | 49.1 | 1.0 (0.4, 2.4) | 10 | 62.5 | 1.7 (0.5, 5.4) | 23 | 65.7 | 2.18 (0.9, 5.3) |
| No | 31 | 43.7 | | 4 | 23.5 | | 58 | 50.9 | | 6 | 37.5 | | 12 | 34.3 | |
| Functional NITAG[2] | | | | | | | | | | | | | | | |
| Yes | 47 | 66.2 | 1.5 (0.7, 3.1) | 14 | 82.4 | 4.0 (1.1, 14.8) | 78 | 68.4 | 5.9 (2.2, 16.0) | 13 | 81.3 | 3.1 (0.8, 11.8) | 23 | 65.7 | 1.2 (0.5, 2.8) |
| No | 24 | 33.8 | | 3 | 17.7 | | 36 | 31.6 | | 3 | 18.8 | | 12 | 34.3 | |
| Eliminated maternal and neonatal tetanus | | | | | | | | | | | | | | | |
| Yes | 71 | 100.0 | n/a | 17 | 100.0 | n/a | 114 | 100.0 | n/a | 16 | 100.0 | n/a | 35 | 100.0 | n/a |
| No | 0 | 0.0 | | 0 | 0.0 | | 0 | 0.0 | | 0 | 0.0 | | 0 | 0.0 | |
| DTP3 coverage ≥95% nationally | | | | | | | | | | | | | | | |
| Yes | 39 | 54.9 | 1.3 (0.6, 2.6) | 7 | 41.2 | 0.9 (0.3, 2.7) | 62 | 54.4 | 1.3 (0.5, 3.0) | 10 | 62.5 | 2.2 (0.7, 7.1) | 18 | 51.4 | 1.0 (0.4, 2.4) |
| No | 32 | 45.1 | | 10 | 58.8 | | 52 | 45.6 | | 6 | 37.5 | | 17 | 48.6 | |
| HIC or UMIC[2] | | | | | | | | | | | | | | | |
| Yes | 64 | 90.1 | 11.3 (4.1,31.8) | 17 | 100.0 | n/a | 97 | 85.8 | 19.4 (7.0, 54.2) | 16 | 100.0 | n/a | 33 | 94.3 | 10.1 (2.0, 50.3) |
| No | 7 | 9.9 | | 0 | 0.0 | | 16 | 14.2 | | 0 | 0.0 | | 2 | 5.7 | |

1. Adjusted for all other characteristics in the table
2. Two countries without world bank income classifications excluded from denominator

## Table S7. Characteristics of WHO-EURO Member States in 2018 With and Without Any Reported Adult Immunization Program

| | Any reported adult vaccination program | | | | | |
|---|---|---|---|---|---|---|
| | Yes N=50 | | No N=3 | | | |
| | Median (IQR) | | Median (IQR) | | p-value[1] | aOR[4] (95%CI) |
| Median per capita health expenditure[2] | 1,616 (795, 3,351) | | 93 (59, 238) | | <0.01 | |
| | n | % | n | % | p-value[3] | |
| World Bank income group | | | | | | |
| Low income | 0 | 0.0 | 1 | 33.3 | <0.001 | 37.3 (1.5, 962.2) |
| Lower-middle income | 5 | 10.0 | 1 | 33.3 | | |
| Upper middle income | 14 | 28.0 | 1 | 33.3 | | |
| High income | 31 | 62.0 | 0 | 0.0 | | |
| Gavi eligible | | | | | | |
| Yes | 0 | 0.0 | 2 | 66.7 | <0.001 | n/a |
| No | 50 | 100.0 | 1 | 33.3 | | |
| Introduced Hepatitis B vaccine birth dose | | | | | | |
| Yes | 38 | 76.0 | 3 | 100.0 | 0.34 | n/a |
| No | 12 | 24.0 | 0 | 0.0 | | |
| Introduced human papilloma virus vaccine | | | | | | |
| Yes | 35 | 70.0 | 0 | 0.0 | 0.01 | n/a |
| No | 15 | 30.0 | 3 | 100.0 | | |
| Introduced rotavirus vaccine | | | | | | |
| Yes | 21 | 42.0 | 1 | 33.3 | 0.78 | 3.4 (0.2, 70.3) |
| No | 29 | 58.0 | 2 | 66.7 | | |
| Functional NITAG | | | | | | |
| Yes | 32 | 64.0 | 2 | 66.7 | 0.93 | 0.5 (0.0, 13.1) |
| No | 18 | 36.0 | 1 | 33.3 | | |
| Third dose diphtheria-tetanus-pertussis coverage ≥95% nationally | | | | | | |
| Yes | 28 | 56.0 | 2 | 66.7 | 0.72 | 0.2 (0.0, 7.3) |
| No | 22 | 44.0 | 1 | 33.3 | | |

[1]p-value for Kruskal-Wallis test for difference in medians
[2]Economic data are from 2016, the most recent year for which such data were available from the World Bank. Data are calculated by the World Bank in International Dollars (Int$), which—by definition—would buy a comparable amount of goods and services in the cited country as the USD would buy in the United States. Excluding 10 countries with missing health care expenditure data
[3]p-value for Chi-square test
[4]Odds ratio adjusted for income category (high or upper middle income vs. lower middle or lower income), having introduced rotavirus vaccine, having a functional NITAG, and having achieved ≥95% national coverage of diphtheria-tetanus-pertussis vaccine
Note: all WHO-EURO countries have achieved the goal of eliminating maternal and neonatal tetanus

**Table S8. Analysis of Immunization Program Characteristics Associated with the Presence of Individual Adult Vaccination Programs in WHO EURO countries**

| | Adult HepB program | | | Adult HZV Program | | | Adult influenza vaccination programme | | | Adult PCV Programme | | | Adult PPSV Programme | | |
|---|---|---|---|---|---|---|---|---|---|---|---|---|---|---|---|
| | Yes N=25 | No N=28 | | Yes N=10 | No N=43 | | Yes N=50 | No N=3 | | Yes N=12 | No N=41 | | Yes N=18 | No N=35 | |
| | n (%) | n (%) | aOR[1] (95% CI) | n (%) | n (%) | aOR[1] (95% CI) | n (%) | n (%) | aOR[2] (95% CI) | n (%) | n (%) | aOR (95% CI) | n (%) | n (%) | aOR (95% CI) |
| Introduced Hepatitis B birth dose | | | | | | | | | | | | | | | |
| Yes | 19 (76.0) | 22 (78.6) | 2.2 (0.5, 10.8) | 7 (70.0) | 34 (79.1) | 0.5 (0.1, 2.7) | 38 (76.0) | 0 (0.0) | n/a | 8 (66.7) | 33 (80.5) | 0.3 (0.0, 1.9) | 11 (61.1) | 30 (85.7) | 0.4 (0.1, 2.1) |
| No | 6 (24.0) | 6 (21.4) | | 3 (30.0) | 9 (20.9) | | 12 (24.0) | 3 (100.0) | | 4 (33.3) | 8 (19.5) | | 7 (38.98) | 5 (14.3) | |
| Introduced HPV | | | | | | | | | | | | | | | |
| Yes | 20 (80.0) | 15 (53.6) | 5.6 (1.3, 24.2) | 10 (100.0) | 25 (58.1) | 2.0 (0.5, 9.1) | 35 (70.0) | 0 (0.0) | n/a | 10 (83.3) | 25 (61.0) | 2.0 (0.3, 12.7) | 17 (94.4) | 18 (51.4) | 11.7 (1.2, 112.4) |
| No | 5 (20.0) | 13 (46.4) | | 0 (0.0) | 18 (41.9) | | 15 (30.0) | 3 (100.0) | | 2 (16.7) | 16 (39.0) | | 1 (5.6) | 17 (48.6) | |
| Introduced Rotavirus vaccine | | | | | | | | | | | | | | | |
| Yes | 10 (40.0) | 12 (42.9) | 0.8 (0.2, 2.9) | 6 (60.0) | 16 (37.2) | 2.6 (0.4, 16.0) | 21 (42.0) | 1 (33.3) | 3.4 (0.2, 70.3) | 7 (58.3) | 15 (36.6) | 2.0 (0.4, 9.0) | 11 (61.1) | 11 (31.4) | 3.6 (0.8, 15.0) |
| No | 15 (60.0) | 16 (57.1) | | 4 (40.0) | 27 (62.8) | | 29 (58.0) | 2 (66.7) | | 5 (41.7) | 26 (63.4) | | 7 (38.9) | 24 (68.6) | |
| Functional NITAG | | | | | | | | | | | | | | | |
| Yes | 14 (56.0) | 20 (71.4) | 0.4 (0.1, 1.5) | 8 (80.0) | 26 (60.5) | 2.6 (0.2, 4.3) | 32 (64.0) | 2 (66.7) | 0.5 (0.0, 13.1) | 10 (83.3) | 24 (58.5) | 5.6 (0.8, 42.4) | 12 (66.7) | 22 (62.9) | 1.0 (0.2, 5.0) |
| No | 11 (44.0) | 8 (28.6) | | 2 (20.0) | 17 (39.5) | | 18 (36.0) | 1 (33.3) | | 2 (16.7) | 17 (41.5) | | 6 (33.3) | 13 (37.1) | |
| DTP3 coverage ≥95% nationally | | | | | | | | | | | | | | | |
| Yes | 16 (64.0) | 14 (50.0) | 1.8 (0.6, 6.1) | 5 (50.0) | 25 (58.1) | 1.0 (0.2, 4.2) | 28 (56.0) | 2 (66.7) | 0.2 (0.0, 7.3) | 8 (66.7) | 22 (53.7) | 3.2 (0.6, 16.3) | 11 (61.1) | 19 (54.3) | 2.2 (0.5, 8.8) |
| No | 9 (36.0) | 14 (50.0) | | 5 (50.0) | 18 (41.9) | | 22 (44.0) | 1 (33.3) | | 4 (33.3) | 19 (46.34) | | 7 (38.98) | 16 (45.7) | |
| HIC or UMIC | | | | | | | | | | | | | | | |
| Yes | 25 (100.0) | 21 (75.0) | n/a | 10 (100.0) | 36 (83.7) | n/a | 45 (90.0) | 1 (33.3) | 37.3 (1.4, 962,2) | 12 (100.0) | 34 (82.9) | n/a | 18 (100.0) | 29 (80.0) | n/a |
| No | 0 (0.0) | 7 (25.0) | | 0 (0.0) | 7 (16.3) | | 5 (10.0) | 2 (66.7) | | 0 (0.0) | 7 (17.1) | | 0 (0.0) | 7 (20.0) | |

[1]Models for adult hepatitis B, zoster, PCV and PPSV vaccination programmes were adjusted for all covariates in the table rows with the exception of country income status
[2]Model for adult influenza vaccination programme was adjusted for all covariates in the table rows with the exception of Hepatitis B birth dose and HPV
